# QTL Mapping of Tiller Number in Korean *Japonica* Rice Varieties

**DOI:** 10.3390/genes14081593

**Published:** 2023-08-06

**Authors:** Dong-Kyung Yoon, Inchan Choi, Yong Jae Won, Yunji Shin, Kyeong-Seong Cheon, Hyoja Oh, Chaewon Lee, Seoyeon Lee, Mi Hyun Cho, Soojin Jun, Yeongtae Kim, Song Lim Kim, Jeongho Baek, HwangWeon Jeong, Jae Il Lyu, Gang-Seob Lee, Kyung-Hwan Kim, Hyeonso Ji

**Affiliations:** 1 Department of Agricultural Biotechnology, National Institute of Agricultural Sciences, Rural Development Administration (RDA), Jeonju 54874, Republic of Korea; dkyoon11@korea.kr (D.-K.Y.); ohhyoja@korea.kr (H.O.); hklee0214@korea.kr (S.L.); chomi1@korea.kr (M.H.C.); jsoojin98@korea.kr (S.J.); koj33@korea.kr (Y.K.); greenksl5405@korea.kr (S.L.K.); firstleon@korea.kr (J.B.); hn36zykyl@korea.kr (H.J.); jaeil99@korea.kr (J.I.L.); kangslee@korea.kr (G.-S.L.); biopiakim@korea.kr (K.-H.K.); 2 Department of Agricultural Engineering, National Institute of Agricultural Sciences, Rural Development Administration (RDA), Jeonju 54875, Republic of Korea; inchchoi@korea.kr; 3 Cheorwon Branch, National Institute of Crop Science, Rural Development Administration (RDA), Cheorwon 24010, Republic of Korea; yjwon@korea.kr; 4 Genecell Biotech Inc., Wanju 55322, Republic of Korea; yunji.shin8@gmail.com; 5 Department of Forest Bioresources, National Institute of Forest Science, Suwon 16631, Republic of Korea; kscheon16@korea.kr; 6 Department of Central Area Crop Science, National Institute of Crop Science, Rural Development Administration (RDA), Suwon 16429, Republic of Korea; wowlek44@korea.kr

**Keywords:** mapping, marker, QTL, rice, tiller number, yield

## Abstract

Tiller number is an important trait associated with yield in rice. Tiller number in Korean *japonica* rice was analyzed under greenhouse conditions in 160 recombinant inbred lines (RILs) derived from a cross between the temperate *japonica* varieties Odae and Unbong40 to identify quantitative trait loci (QTLs). A genetic map comprising 239 kompetitive allele-specific PCR (KASP) and 57 cleaved amplified polymorphic sequence markers was constructed. *qTN3*, a major QTL for tiller number, was identified at 132.4 cm on chromosome 3. This QTL was also detected under field conditions in a backcross population; thus, *qTN3* was stable across generations and environments. *qTN3* co-located with QTLs associated with panicle number per plant and culm diameter, indicating it had pleiotropic effects. The *qTN3* regions of Odae and Unbong40 differed in a known functional variant (4 bp TGTG insertion/deletion) in the 5ʹ UTR of *OsTB1*, a gene underlying variation in tiller number and culm strength. Investigation of variation in genotype and tiller number revealed that varieties with the insertion genotype had lower tiller numbers than those with the reference genotype. A high-resolution melting marker was developed to enable efficient marker-assisted selection. The QTL *qTN3* will therefore be useful in breeding programs developing *japonica* varieties with optimal tiller numbers for increased yield.

## 1. Introduction

Rice (*Oryza sativa*) is one of the most important food crops in the world. According to FAOSTAT, 787 million tons of rice were produced in 2021, surpassing wheat production [1]. Over the next 30–40 years, it is anticipated that an additional 200 million tons of rice will need to be produced in order to meet the rising demand [2]. This will require the development of higher yielding varieties that require less fertilizer and are resistant to the intensifying biotic and abiotic stresses resulting from global climate change.

The modification of plant architecture by ideotype breeding has increased crop yield potential [3]. In order to improve rice yield, a new plant type was conceptualized in 1988 [4]. It is difficult to increase the yield of modern semi-dwarf rice varieties because they have a large number of unproductive tillers and excessive leaves, which cause shading, resulting in reduced canopy photosynthesis and sink size [3]. Therefore, the International Rice Research Institute (IRRI) proposed the “New Plant Type (NPT)”, a plant with modified architecture, including low tillering capacity and no unproductive tillers. 

Rice tillering is an essential agronomic trait as it determines the number of panicles per plant, which is a fundamental component of rice grain yield [5,6,7]. A large number of tillers may diminish the final yield because they consume an excessive amount of nutrients from the main branch during the vegetative stage and senesce before maturity [8]. Tiller morphology is also important in crop adaptability and phenotypic plasticity [8]. “Phenotypic plasticity” refers to all the morphological, physiological, and phenological changes in the phenotype of a given genotype in response to environmental changes [9]. Tiller development is controlled by an intricate network of genetic, hormonal, and environmental influences, making tillering a highly flexible trait that enables wild cereals to adapt to diverse environmental conditions [5,10]. High tiller numbers are preferred under optimal growth conditions, but low tiller numbers are better adapted to severe environments [11]. Thus, regulation of tiller number is an important target in breeding programs that aim to modify plant architecture. According to Sakamoto and Matsuoka [12] and Makela and Muurinen [13], it is necessary to achieve a balance between tiller number and vigor. Although tillers can produce inflorescences, and thus contribute to grain yield, those that develop late in the season do not produce any grain and thus lower the overall harvest index [12,13]. A model plant with a low number of unproductive tillers, high number of grains per panicle, reduced height, and erect leaves was proposed as the ideotype for cereal breeding [3]. Before the Green Revolution, rice varieties responded to nitrogen fertilizer by abundant tillering and stem elongation, thus increasing biomass. Ideotype breeding produced shorter, more productive plants with an improved response to nitrogen, increasing the harvest index to 0.5 [12]. This ideotype is associated with particular alleles at the locus *wealthy farmer’s panicle (wfp)/ideal plant architecture 1 (ipa1)*, which reduce tillering and induce the desired inflorescence characteristics that lead to increased yield [10].

Rice yield is a complex characteristic that is heavily impacted by many environmental conditions and regulated by numerous genes within quantitative trait loci (QTLs) [12,14]. QTL mapping with molecular markers is an effective strategy for analyzing complex traits in crops. Better understanding of the genetic foundation of the quantitative traits utilized in plant breeding via QTL mapping is a significant research area in developmental quantitative genetics [15,16]. The genetic and molecular mechanisms that control tiller number in rice have been studied for several decades [15,17,18,19]. The Gramene Database (http://archive.gramene.org/ (accessed on 20 March 2023)) contains details of 213 QTLs reported to control tiller number in rice. Yan et al. [20] mapped a significant QTL for tiller number on rice chromosome 1. Cui et al. [21] suggested that pleiotropic effects of chromosomal regions and two-locus combinations may be the genetic basis underlying the relationships between heading date, tiller number, and plant height. Liu et al. [19] detected 14 QTLs that significantly affected rice tillering using the conditional analysis method in a single-segment substitution population of rice. A genome-wide association study (GWAS) identified 38 QTLs associated with effective tiller number (ETN), of which 4 colocalized with *NAL1, OsWRKY74, OsAAP1,* and *DWL2*; this showed that *Hap5* of *OsAAP1, Hap3* and *Hap6* of *DWL2*, and *Hap3* and *Hap4* of *WRKY74*, are desirable alleles that control tiller number effectively and are involved in the regulation of rice tillering [22]. Another recent GWAS identified 23 loci associated with variation in tiller number (LATNs) [23]. In addition, great progress has been made using mutants to identify key genes regulating tiller number in rice. Since the cloning of *MOC1*, the key gene regulating tiller number [24], many other genes affecting tiller number have been discovered through mutant analysis, including *MOC3* [25], *FON1* [26], *DLT* [27], and others [28]. The formation of a rice tiller can be divided into two processes (initiation of axillary meristem (AM) and its growth) and the genes involved in these processes were well reviewed by Yan et al. [29]. Regulator genes influencing AM formations include *OSH1, LAX1, LAX2, MOC1, MIP1, TAD1, SLR1, MOC3, RFL, CUC1* and *FZP,* while regulator genes influencing AM growth into tillers include *D3, D10, D14, HTD1, D27, OsMAX1a, OsMAX1e, D53, OsMADS57, OsTB1, IPA1, DEP1, OsSHI1, OsCCA1, Hd3a, OsDRM2, OsWRKY94, FON1, TN1,* and *TIF1* [29]. Plant hormones acted in controlling axillary bud growth and tiller number. Auxin, strigolactones, and gibberellins negatively regulated the formation of rice tillers, whereas brassinosteroids and cytokinins positively regulated tiller formation, and many genes in hormone regulation pathway such as *OSPIN1, OsIAA6, OsCKX2, GID1, OsBZR1*, etc., influence tiller bud growth [29]. However, our current understanding of rice tillering regulation mechanisms is still limited [29] and we need to identify more QTL genes for tillering in rice.

Methane is one of the greenhouse gases causing increase in global surface temperature, and rice paddy fields are known to be a major anthropogenic source of atmospheric methane [30]. Rice plants may exert significant impacts on methane emissions in paddy fields, and some aboveground morphological traits of rice, such as plant height, tiller number and leaf area, have been shown to be positively correlated with soil methane emissions [31]. A few studies reported positive correlation between tiller number and methane emission [30,32,33,34]. Therefore, it would be probable that rice varieties with lower numbers of tillers may also emit lower quantities of methane.

We previously developed a population of recombinant inbred lines (RILs) from a cross between Odae, a variety which is resistant to pre-harvest sprouting (PHS), and Unbong40, which is susceptible to PHS. We identified five QTLs for PHS in this population [35]. At that time, we noticed that the parental lines differed in plant type, as Odae had a high tiller number and Unbong40 a low tiller number. In the current study, we aimed to find QTLs for tiller number with this RIL population. A major QTL was found on chromosome 3 and its candidate gene was proposed to be *OsTB1*. A selection marker for this QTL was also developed. These results will provide an effective tool for developing Korean *japonica* rice varieties with an optimal tiller number for increased yield and potential for reduced methane emission. 

## 2. Materials and Methods

### 2.1. Plant Growth and DNA Extraction

In total, 160 RILs from a population derived from a cross between Odae and Unbong40 were cultivated over two periods in 2019/2020 and 2022 in a greenhouse; at these times, the RILs were in the F_9_ and F_10_ generations, respectively. All RILs and the two parental varieties were grown in a greenhouse (maximum/minimum temperatures: 32 °C/22 °C; and light/dark periods: 14 h/10 h). Seeds of all lines were sown in early December in 2019 and in May 2022 in 200-well growth trays. Seedlings were transplanted to pots (140 mm diameter) three weeks after sowing. The spaces between plants were 26 × 52 cm in 2019 and 20 × 20 cm in 2022. In addition, Odae^*5^/Unbong40 BC_4_F_2_ and BC_4_F_3_ and backcross populations were cultivated in 2021 and 2022, respectively, in an experimental field at the National Institute of Agricultural Sciences of the Rural Development Administration (Jeonju, Republic of Korea). In field trials, seeds of the two parental varieties and BC_4_F_2_ or BC_4_F_3_ were sown in mid-May of both years, and seedlings were transplanted to ensure 30 × 15 cm spacing between the plants in early June. Genomic DNA was extracted using a Plant gDNA Extraction Kit (Biomedic, Bucheon, Republic of Korea). 

### 2.2. Phenotypic Evaluations

In the 2019/2020 greenhouse experiment, the tiller numbers of plants in the F_9_ RIL population from a cross between Odae and Unbong40 were counted 41 and 47 days after sowing (DAS); in the 2022 greenhouse experiment, tiller number in the F_10_ RIL population was counted at weekly intervals between 40 and 61 DAS. In addition, other major agronomic traits, including culm length (CL), panicle length (PL), culm diameter (CD), panicle number per plant (PN), and grain number per panicle (GNP) were measured in the F_10_ RIL population at harvest in 2022 greenhouse experiment. CL, PL, and CD were measured in the main culm of plants that had been labeled with colored tape at the early tillering stage. All measurements for TN, CL, PL, CD, PN, and GNP were made using five replicates per line. Moreover, 192 BC_4_F_3_ lines (20 plants per line) derived from each ancestral BC_4_F_2_ plant were grown in the field and the TN of 15 BC_4_F_3_ plants from each line was measured at 64 and 71 DAS, with the mean value being considered to be the TN of the ancestral BC_4_F_2_ plant.

### 2.3. Mapping and Identification of QTL

A genetic map comprising of 239 kompetitive allele-specific PCR (KASP) and 49 cleaved amplified polymorphic sequence markers (CAPS), constructed using data from 160 F_9_ RILs derived from a cross between Odae and Unbong40 [35], was used for QTL analysis. After determining that a major QTL for tiller number was located on chromosome 3, we developed eight additional CAPS markers based on sequence variation discovered by analyzing the genome resequencing data from Odae and Unbong40. Genome resequencing data analysis was performed using the method described previously by Ji, et al. [36]. CAPS markers were designed using the methodology described in Cheon, et al. [37]. Finally, a revised genetic map comprised of 294 markers, including 239 KASP and 58 CAPS markers, was constructed using the MapDisto 1.7 program [38], together with the MapChart program [39]. QTL analysis was performed via composite interval mapping (CIM) using the Windows QTL Cartographer ver. 2.5 program [40]. The logarithm of the odds (LOD) threshold was calculated through 1000× permutations with a probability level of 0.05. CIM was performed using the default conditions of the Windows QTL Cartographer ver. 2.5 program.

### 2.4. Analysis of OsTB1, a Candidate Gene for qTN3 on Chromosome 3

To clarify sequence variation in *OsTB1*, a gene on chromosome 3 that was a strong candidate for *qTN3*, the major QTL for tiller number, we tested 54 Korean *japonica* rice varieties, including the parental varieties Odae and Unbong40. These varieties were cultivated in a greenhouse. Seeds were sown in 200-well growth trays in late December 2022 and seedlings were transplanted to pots (140 mm diameter) 3 weeks after sowing. The spacing between plants was 20 × 20 cm. Tiller number was counted at 41 and 47 DAS using three replicates per variety. DNA was extracted from fresh leaves using a DNeasy Plant mini kit (Qiagen, Hilden, Germany). *OsTB1* gene fragments were amplified from genomic DNA using specific primer pairs designed according to the corresponding gene sequences deposited in The Rice Annotation Project database (RAP-DB; https://rapdb.dna.affrc.go.jp/index.html, accessed on 25 October 2022)). All primer sequences are listed in Supplementary Table S1. Amplified PCR products were cleaned using ExoAP enzyme (EZ^TM^ ExoAP PCR Product Clean-up Mix, Enzynomics, Daejeon, Republic of Korea) before sequencing. DNA sequences were analyzed using the CLC Genomics Workbench 6 (Qiagen, Hilden, Germany) program.

We developed a high-resolution melting (HRM) marker to study sequence variation in *OsTB1* and used this marker to genotype all 54 rice varieties (Supplementary Table S1). For genotyping, 2.5 μL sample DNA was added to SsoFast EvaGreen Supermix (BIO-RAD, Hercules, CA, USA) to obtain a 20 μL final reaction volume. PCR amplifications were performed in a CFX Connect Thermal Cycler (BIO-RAD, Hercules, CA, USA) using the following protocol: 40 cycles of denaturation at 95 °C for 5 s and annealing/extension at 58 °C for 20 s. HRM analysis was performed following PCR amplification using the manufacturer’s recommended settings for temperature ramping and fluorescence acquisition; that is, temperature ramping from 65 to 95 °C, rising by 0.2 °C/1 s. The melting curves were normalized in the 78.1 to 84.0 °C normalization regions before and after the major fluorescence decrease, which represented the melting of the PCR product, using Precision Melt Analysis software (BIO-RAD, Hercules, CA, USA). All samples were plotted according to their melting profiles. 

## 3. Results

### 3.1. Analysis of Phenotypic Variation

**Figure 1:**
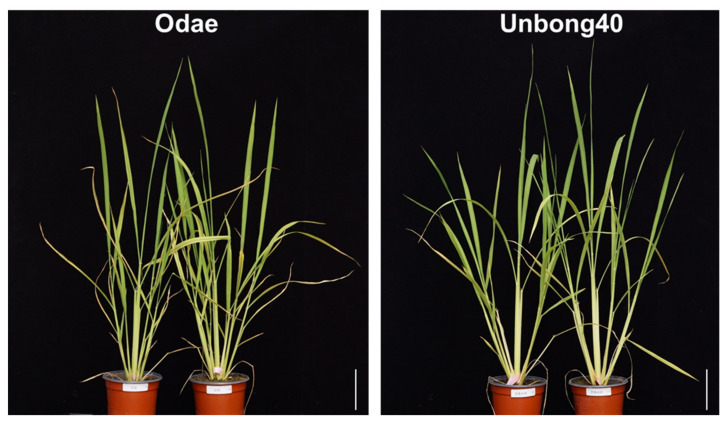
Phenotypes of the parental varieties Odae and Unbong40 photographed 32 days after sowing (DAS). Scale bars = 10 cm.

**Figure 2:**
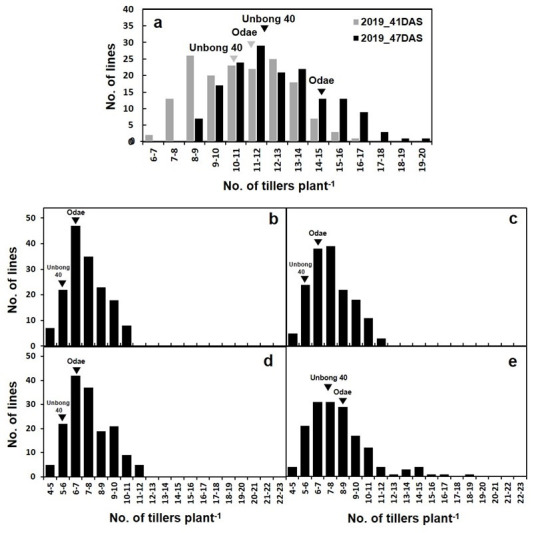
Distributions of tiller number in the RIL population derived from a cross between Odae and Unbong40. Tiller number was measured in (**a**) the F_9_ population grown in 2019 at 41 and 47 DAS and (**b**–**e**) in the F_10_ population grown in 2022 at (**b**) 40 DAS, (**c**) 47 DAS, (**d**) 54 DAS, and (**e**) 61 DAS. (**a**–**e**) Inverted triangles indicate the parental varieties Odae and Unbong40.

To investigate phenotypic variation in the F_9_ RIL population, we measured TN at 41 and 47 DAS in the 2019/2020 greenhouse experiment. Figure 1 shows the phenotypes of the parental varieties, Odae, which has high TN, and Unbong40, which has low TN. At 41 DAS, the mean TN of Odae and Unbong40 was 11.3 and 11.0, respectively, and, among the RILs, the class TN 8-9 had the highest frequency (Figure 2a). By 47 DAS, the mean TN of Odae and Unbong40 was 14.3 and 12.0, respectively, and the class TN 11-12 had the highest frequency (Figure 2a). 

In the 2022 greenhouse experiment, the mean TNs of Odae, measured weekly between 40 and 61 DAS, were 6.4, 6.4, 6.6, and 8.6, respectively, while the mean TNs of Unbong40 were 5.4, 5.4, 5.4, and 7.2, respectively. Among the F_10_ RIL population grown in 2022, the class TN 6-7 had the highest frequency at 40 DAS and the class TN 7-8 at 47 DAS, whereas the class TN 6-7 had the highest frequency at both 54 and 61 DAS (Figure 2b–e).

### 3.2. QTL Mapping of Tiller Number

**Figure 3:**
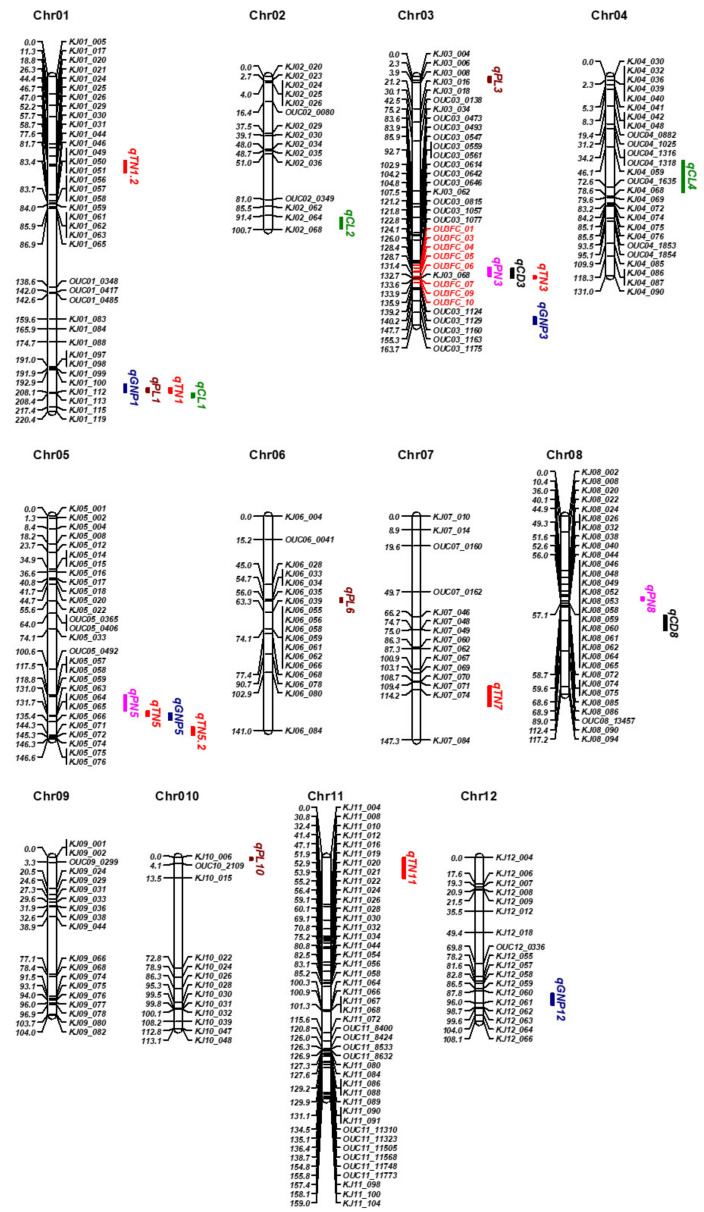
Genetic map showing the locations of QTLs associated with tiller number and other agronomic traits in the population of 160 RILs derived from a cross between Odae and Unbong40. Chromosome numbers are shown at the top of each chromosome. Marker names are listed on the right side of each chromosome, and the genetic distance of each marker from the first marker at the top of a chromosome is shown on the left side. The eight markers on chromosome 3 that were developed in this study are marked in red. The positions of QTLs (vertical text) are indicated on the far-right side of each chromosome. Red, black, pink, indigo, and green color symbols indicate names of QTLs for tiller number, culm diameter, panicle number, grain number for panicle, and culm length, respectively.

In our previous study, we used data from the F_9_ RIL population derived from a cross between Odae and Unbong40 to construct a genetic map comprising 288 DNA markers, including 239 kompetitive allele-specific PCR (KASP) and 49 cleaved amplified polymorphic sequence (CAPS) markers. In the current study, we performed a QTL analysis using this genetic map and TN data from the F_9_ RIL population. This analysis identified a major QTL for TN near the marker *KJ03_068* on rice chromosome 3. Sequence variation between the parental lines Odae and Unbon40, revealed by a resequencing data analysis, enabled us to develop eight additional CAPS markers in the vicinity of *KJ03_068*, which were used to genotype the RIL population. This enabled the construction of a revised genetic map for the RIL population comprising 296 DNA markers (239 KASP and 58 CAPS markers; Figure 3). This revised map had a total length of 1652.1 cm and a mean interval between markers of 5.98 cm.

Using this revised genetic map and the TN data from the F_9_ RIL population, we identified a major QTL for tiller number. This QTL was present at both 41 and 47 DAS in the 2019 data with LOD scores of 38.6 and 32.8, respectively. We named this QTL *qTN3* (Table 1; Supplementary Figure S1a). The additive effects of *qTN3* at 41 DAS and 47 DAS were 1.56 and 1.53, respectively, with the Odae-type allele increasing TN; its *R^2^* values were 0.475 and 0.402, respectively. *qTN3* was located in a 1.4 cm interval between the markers *OU3FC_05* and *KJ03_068*. 

The presence of *qTN3* was verified in 2022 through repeated experiments in a greenhouse using the F_10_ RIL population. In 2022, TN measurements were collected at weekly intervals from 40 DAS to 61 DAS, and *qTN3* was detected at the same position on chromosome 3 in all four datasets (Supplementary Figure S1a). At 40 DAS, the LOD score, additive effect, and *R^2^* value for *qTN3* were 25.3, 0.88, and 0.335, respectively, and, at 47 DAS, the LOD score, additive effect, and *R^2^* value of *qTN3* were 23.9, 0.91, and 0.296, respectively; later, at 54 DAS, the LOD score, additive effect, and *R^2^* value of *qTN3* were 24.7, 0.93, and 0.302, respectively; and, finally, at 61 DAS, the LOD score, additive effect, and *R^2^* value of *qTN3* were 21.6, 1.12, and 0.188, respectively. The analysis revealed other QTLs affecting TN. *qTN1* was identified at 208.1 cm on chromosome 1; this QTL was also detected in the 41 DAS dataset in 2019. A further two QTLs, *qTN5* (LOD score 5.5) and *qTN7* (LOD 5.0), were identified in the 40 DAS data collected in 2022. *qTN5* (LOD 5.6) and *qTN7* (LOD 4.7) were located at 130.8 cm on chromosome 5 and at 111.4 cm on chromosome 7, respectively. Analysis of data collected at 54 DAS identified *qTN5* on chromosome 5, *qTN7* on chromosome 7, and another QTL, *qTN11*, on chromosome 11. In addition, analysis of the 61 DAS data identified *qTN5* with a LOD score of 6.0 at 131.0 cm on chromosome 5.

To confirm the effects of *qTN3*, we produced 15 Odae^*5^/Unbong40 BC_4_F_1_ plants and genotyped them using 180 KASP markers distributed over 12 rice chromosomes. We selected OU3-32, the BC_4_F_1_ plant with the highest recurrent parent genome recovery rate (94.4%) and cultivated 196 BC_4_F_2_ offspring derived from this plant (Supplementary Figure S2a,b). We genotyped all 196 plants using 14 markers on chromosome 3. In 2022, 192 BC_4_F_3_ lines (20 plants per line) derived from each ancestral F_2_ plant were grown in the field. The TN of 15 BC_4_F_3_ plants from each line was measured at 64 and 71 DAS, and the mean value was considered to be the TN of the ancestral BC_4_F_2_ plant. A QTL analysis was performed by integrating the genotype data and TN values of the 196 BC_4_F_2_ plants. This analysis identified *qTN3* near the marker *KJ03_068* with LOD scores of 11.5 and 6.0 at 64 and 71 DAS, respectively (Supplementary Figure S2b; Supplementary Table S1b). These results suggested that *qTN3* was very stable across generations and in different environments. 

### 3.3. QTL Mapping for Some other Yield-Related Traits

We measured other agronomic traits related to grain yield, including CL, PL, CD, PN, and GNP, in the F_10_ RIL population in the 2022 greenhouse experiment, and identified QTLs associated with those traits (Figure 3; Table 2; Supplementary Figure S3a–e). Our analysis identified three QTLs for CL on chromosomes 1, 2, and 4 with LOD scores of 27.0, 4.4, and 3.3, respectively. We found four QTLs for PL on chromosomes 1, 3, 6, and 10; their LOD scores were 6.9, 4.0, 5.6, and 4.0, respectively. There were three QTLs for PN on chromosomes 3, 5, and 8 with LOD scores of 4.9, 3.3, and 3.4, respectively. Two QTLs for CD were identified on chromosomes 3 (qCD3) and 8 (qCD8), with LOD scores of 4.2 and 6.3, respectively. Finally, four QTLs associated with GNP were detected on chromosomes 1 (qGNP1), 3 (qGNP3), 5 (qGNP5), and 12 (qGNP12); the LOD scores of these QTLs ranged from 4.5 to 9.9. qTN3 co-located with qPN3 and qCD3, which suggested that the gene responsible for qTN3 may have pleiotropic effects on TN, PN, and CD.

### 3.4. Analysis of Candidate Genes for the QTL qTN3

The greenhouse experiments conducted in 2019/2020 and 2022 identified *qTN3*, a QTL located between the markers *OU3FC_05* and *OU3FC_07* (Table 1), which corresponded to a 376 kbp region between 28,354,128 and 28,730,425 bp. We analyzed whole-genome resequencing data from Odae and Unbong40 previously generated using the Illumina Hiseq sequencing platform [35] and found that the *qTN3* region contained 86 sequence variations between Odae and Unbong40; these are listed in Supplementary Table S3. These variations were located in sequences upstream and downstream of genes, in intergenic regions, in introns, and in the 5ʹ and 3ʹ untranslated regions (UTR) of 10 genes (Supplementary Table S3). Of the 35 genes listed in the Supplementary Table S3, 2 (Os03g0705300 and Os03g0706500) have been previously associated with tillering. Os03g0705300, also known as *OsPIP5K1*, interacts with *DWT1* and controls uniform growth of the main shoot and tillers [41]. All six of the sequence variations found between Odae and Unbong40 in this gene region were located upstream of this gene and therefore seemed unlikely to affect gene function and phenotype. The Os03g0706500 sequences of Odae and Unbong40 contained two variants: a G/A SNP in the upstream region and a TGTG insertion/deletion in the 5ʹ UTR. Os03g0706500 is also known as *RICE TEOSINTE BRANCH1 (OsTB1), FINE CULM1 (FC1),* and *STRONG CULM 3 (SCM3*; it encodes a TCP family transcription factor that acts downstream of strigolactone signaling to inhibit outgrowth of the axillary buds in rice) [42,43,44,45]. This TGTG insertion/deletion has been previously shown to cause changes in *OsTB1* expression, resulting in variation in tiller number, CD, and GNP [21,44,45]. In this context, it is notable that the Unbong40 sequence contains the TGTG insertion but that of Odae does not, making it highly probable that this difference is the genetic factor responsible for the variation between Odae and Unbong40 in TN, PN, and CD, and that *OsTB1/FC1/SCM3* is the gene underlying the effects of the *qTN3* QTL.

**Figure 4:**
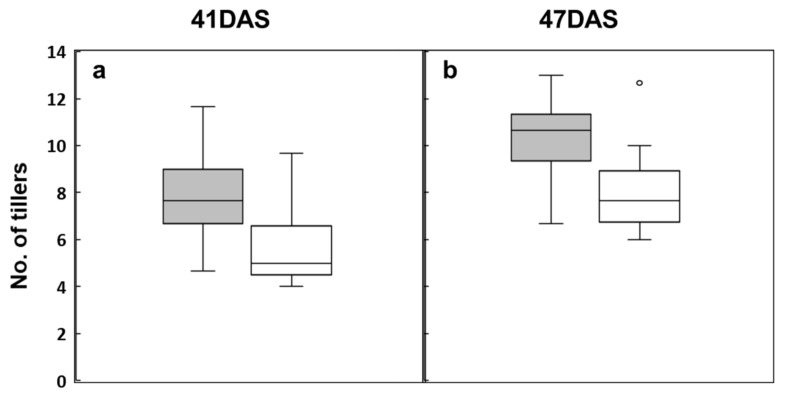
Boxplot showing the distribution tiller number in 54 Korean japonica rice varieties classified by OsTB1 genotype. Tiller numbers were measured at (**a**) 41 DAS and (**b**) 47 DAS. Gray boxes: reference genotype; white boxes: TGTG insertion genotype.

To investigate the relationship between the TN phenotype and the TGTG insertion genotype in *OsTB1*, we used Sanger sequencing to determine sequence variation within 54 Korean *japonica* rice cultivars, including Odae and Unbong40; we also counted TN at 41 and 47 DAS in the same cultivars (Figure 4). We found that 13 varieties including Unbong40 contained the TGTG insertion and that the remaining 41 varieties, including Odae, possessed the reference genotype that lacks the insertion (Supplementary Table S4). Figure 4 shows the distribution of TN between the varieties according to their genotype at the TGTG insertion site. At both 41 DAS (Figure 4a) and 47 DAS (Figure 4b), the number of tillers was lower in cultivars with the TGTG insertion genotype than in cultivars with the reference genotype.

**Figure 5:**
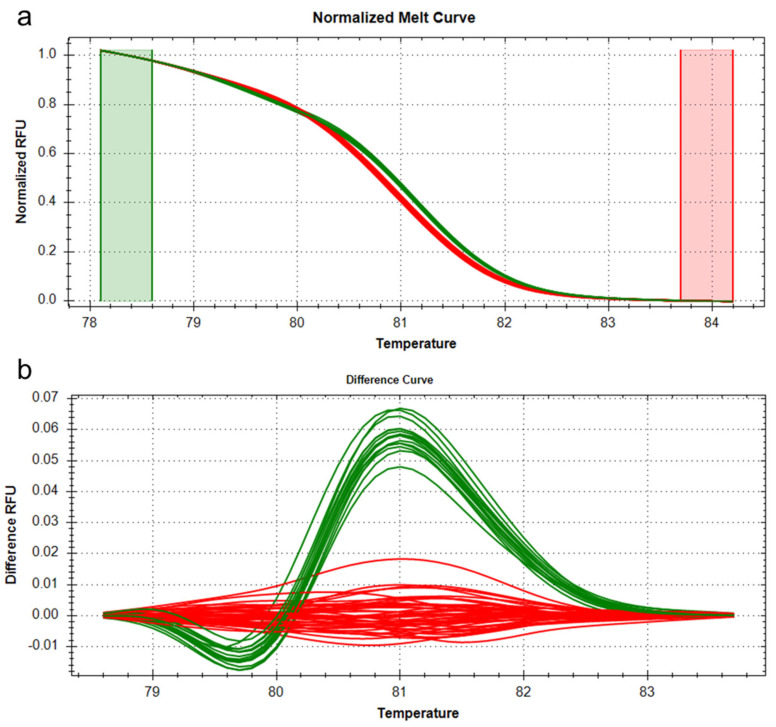
Analysis of HRM data from 54 Korean *japonica* varieties. (**a**) Normalized melt curve plots and (**b**) differential curve plots of internal amplicons using the HRM marker (88 bp amplicon). The melting curves of varieties with the reference genotype and the alternative insertion genotype are shown in each plot. Red: reference genotype; green: 4 bp TGTG insertion genotype.

In addition, we developed a novel DNA marker to identify both sequence variants by HRM technology (Supplementary Table S1) and used the melting profile of this HRM marker to genotype all 54 varieties (Figure 5). This analysis revealed clear differences between varieties with the TGTG insertion genotype varieties and those with the reference genotype. Taken together, all these results demonstrate that the presence or absence of the TGTG insertion in OsTB1 may be responsible for differences in TN within Korean japonica rice varieties and that the novel HRM marker developed in this study will be a useful tool for genotyping variation at this locus.

## 4. Discussion

Tillering is one of the most important factors determining grain yield in rice [7,46]. For several decades, scientists have tried to identify and characterize QTLs associated with TN; however, despite numerous remarkable accomplishments, many of the genes responsible for tiller-related QTL remain unidentified. Moreover, grain yield is a complex quantitative trait affected by environmental factors as well as various genes. Identification of additional QTLs affecting TN and yield is essential for a better understanding of the genetic basis underlying grain yield traits. 

We identified *qTN3,* a major QTL for TN, by its very high LOD score (38.6). *qTN3* was located in a region between 28,354,128 bp and 28,730,425 bp on chromosome 3 and its effects were very stable across generations and under different growth conditions. A comparison of genomic sequences from Odae and Unbong40 revealed that these varieties differed in a 4 bp TGTG insertion/deletion in Os03g0706500 (also known as *OsTB1*, *SCM3*, and *FC1*). Takeda et al. (2003) [44] demonstrated that transgenic rice plants over-expressing *OsTB1* exhibit reduced TN, whereas a loss of-function mutation in *OsTB1* results in increased tillering. *SCM3* negatively regulates TN and positively regulates culm strength and spikelet number [45]. Use of CRISPR/Cas9 gene editing to insert TGTG into the *OsTB1* 5’ UTR enhances gene expression, consequently increasing stem cross-section area (SCSA) and decreasing TN per plant; this insertion is naturally present in varieties with a large SCSA [21]. It was therefore highly likely that *OsTB1* was the gene underlying the effects of *qTN3,* and the 4 bp TGTG insertion/deletion was the causative variation responsible for differences in TN between Odae and Unbong40. *qTN3* colocalized with *qPN3*, a QTL associated with PN, and also with *qCD3*, a QTL affecting CD, consistent with previous reports that *OsTB1* controls these traits [45]. The pleiotropic effects of *OsTB1* are an important observation from the viewpoint of breeding, since both TN and PN directly govern grain yield, whereas CD is important in lodging resistance, another valuable grain yield trait [45]. Investigation of sequence variation within 54 Korean *japonica* rice varieties revealed that varieties with the 4 bp TGTG insertion genotype had a lower TN (Figure 4; Supplementary Table S4). In summary, we identified a very effective allele of *OsTB1* that reduced TN and increased culm strength in Korean *japonica* rice varieties. This allele may be useful in ideotype breeding to obtain varieties with a low number of unproductive tillers. In addition, we developed a novel HRM marker to enable genotyping of variation at this locus, which will facilitate use of this allele in marker-assisted selection. Recently, it was revealed that an *OsTB1* duplicate gene, *OsTB2*, has been artificially selected during upland rice adaptation and that natural variation in *OsTB2* is associated with tiller number [47]. Moreover, a novel allele of *IPA1* gene, *IPA1-2D*, resulted in greater yield increase than IPA1 [48]. It would be desirable to try to combine alleles of genes controlling tiller number, culm strength, and panicle size such as *OsTB1, OsTB2, and IPA1* to develop better ideal plant type rice varieties. 

Meanwhile, methane is an extremely potent greenhouse gas that is accelerating climate change. Methane emissions from paddy fields are a major environmental consequence of rice cultivation. For this reason, various efforts are being made to reduce the amount of methane produced by growing rice. Several important plant physiological parameters, including leaf number, TN, and plant biomass, were proposed to be important factors regulating methane emissions from rice plants. Modifying these physiological traits may enable rice breeders to develop new varieties emitting lower levels of methane. TN was reported to be positively related to methane emission rates during the tillering stage [33]. Tiller numbers were linearly related to methane transport capacity (MTC) of rice plants in 12 cultivars [32] and 22 cultivars [30], and plants of NPT and KDML 105 cultivars that had the minimum number of tillers and smaller biomass exhibited low MTC [30]. Therefore, rice varieties with lower numbers of tillers may also emit lower quantities of methane. Our results, which enable a reduction in the number of tillers, may therefore also provide a potential method for reducing methane emissions in rice cultivation.

By identifying QTL associated with TN, this study provides a strong foundation for the development of ideotype breeding programs aimed at improving grain yield and lodging resistance in Korean *japonica* rice cultivation, along with possible reductions in methane emissions.

## 5. Conclusions

We performed QTL mapping for tiller number with the RIL population derived from a cross between Odae and Unbong40 and identified a major QTL, *qTN3*, for tiller number on chromosome 3. The effects of this QTL were stable across different generations and environmental conditions. We also found that *qTN3* co-located with QTLs associated with panicle number per plant and culm diameter, indicating that it had pleiotropic effects on those traits. We found a known functional variant (4 bp TGTG insertion/deletion) in the 5ʹ UTR of *OsTB1*, a gene underlying variation in tiller number and culm strength, in the *qTN3* regions of Odae and Unbong40 through genome sequencing data analysis. In 54 Korean *japonica* rice varieties, varieties with the insertion genotype had lower tiller numbers than those with the reference genotype. We developed a high-resolution melting marker for this variation to enable efficient marker-assisted selection. These results provide an effective tool for developing Korean *japonica* rice varieties with an optimal tiller number for increased yield and possibly reduced methane emission.

## Figures and Tables

**Table 1:** Quantitative trait loci affecting tiller number identified in the RIL population derived from a cross between the *japonica* varieties, Odae and Unbong40.

Measurement Date (DAS)	QTL Name	Chr.	Location (cm)	QTL Interval (cm)	Interval-Flanking Markers		LOD	Additive Effect	*R^2^*
					Left	Right			
14 March 2019 (41)	*qTN1*	1	208.1	204.9–208.3	KJ01_100	KJ01_113	4.9	0.42	0.036
	*qTN3*	3	132.4	131.3–132.6	OU3FC_05	KJ03_068	38.6	1.56	0.475
	*qTN7*	7	117.2	111.9–125.3	KJ07_071	KJ07_084	8.5	−0.65	0.083
20 March 2019 (47)	*qTN1.2*	1	57.7	55.1–63.1	KJ01_029	KJ01_044	5.7	−0.54	0.046
	*qTN3*	3	132.4	131.3–132.9	OU3FC_05	OU3FC_07	32.8	1.53	0.402
	*qTN5.2*	5	142.4	138.7–144.3	KJ05_066	KJ05_071	4.3	−0.49	0.043
	*qTN7*	7	117.2	114.2–124.1	KJ07_074	KJ07_084	7.7	−0.70	0.082
10 June 2022 (40)	*qTN1*	1	208.1	204.5–208.4	KJ01_112	KJ01_113	4.7	0.31	0.045
	*qTN3*	3	132.4	131.2–132.6	OU3FC_06	KJ03_068	25.3	0.88	0.335
	*qTN5*	5	129.8	125.1–132.1	KJ05_059	KJ05_066	5.5	−0.37	0.060
	*qTN7*	7	112.4	109.4–120.2	KJ07_071	KJ07_084	5.0	−0.36	0.054
17 June 2022 (47)	*qTN3*	3	132.4	131.1–132.6	OU3FC_06	KJ03_068	23.9	0.91	0.296
	*qTN5*	5	130.8	125.7–131.7	KJ05_059	KJ05_066	5.6	−0.40	0.060
	*qTN7*	7	111.4	106.2–121.5	KJ07_069	KJ07_084	4.7	−0.37	0.046
24 June 2022 (54)	*qTN3*	3	132.4	131.1–132.5	OU3FC_06	KJ03_068	24.7	0.93	0.302
	*qTN5*	5	130.8	125.3–131.7	KJ05_059	KJ05_066	5.1	−0.39	0.054
	*qTN7*	7	111.4	105.8–121.8	KJ07_069	KJ07_084	4.4	−0.36	0.042
	*qTN11*	11	3.0	0–14.0	KJ11_004	KJ11_008	3.4	0.33	0.040
1 July 2022 (61)	*qTN3*	3	132.4	131.2–132.6	OU3FC_06	KJ03_068	21.6	1.12	0.188
	*qTN5*	5	131.0	128.3–131.9	KJ05_059	KJ05_066	6.0	−0.70	0.078

DAS: days after sowing; Chr.: chromosome number; QTL Interval: region containing the QTL at 95% probability; LOD: logarithm of the odds score.

**Table 2:** QTLs associated with different agronomic traits identified in the F_10_ RIL population derived from a cross between Odae and Unbong40.

Trait	QTL Name	Chr.	Location (cm)	QTL Interval (cm)	Interval-Flanking Markers		LOD	Additive Effect	*R^2^*
					Left	Right			
CL	*qCL1*	1	209.4	208.4–211.5	KJ01_113	KJ01_115	27.0	−5.26	0.437
	*qCL2*	2	99.4	92.7–100.4	KJ02_064	KJ02_068	4.4	−1.73	0.048
	*qCL4*	4	66.1	55.3–76.1	KJ04_059	OUC04_1635	3.3	1.94	0.058
PL	*qPL1*	1	207.9	204.9–208.1	KJ01_099	KJ01_112	6.9	−0.65	0.110
	*qPL3*	3	2.0	0–3.9	KJ03_004	KJ03_008	4.0	−0.48	0.059
	*qPL6*	6	54.7	54.0–56.7	KJ06_028	KJ06_039	5.6	0.60	0.097
	*qPL10*	10	0.0	0–2.0	KJ10_006	OUC10_2109	4.0	0.49	0.059
PN	*qPN3*	3	128.7	125.5–131.4	OU3FC_01	KJ03_068	4.9	0.39	0.096
	*qPN5*	5	122.8	117.5–128.2	KJ05_058	KJ05_063	3.3	−0.37	0.092
	*qPN8*	8	54.6	53.4–55.6	KJ08_040	KJ08_044	3.4	1.86	0.119
CD	*qCD3*	3	130.7	126.3–132.7	OU3FC_03	OU3FC_07	4.2	−0.12	0.071
	*qCD8*	8	67.7	65.4–75.5	KJ08_075	OUC08_13457	6.3	−0.17	0.144
GNP	*qGNP1*	1	207.9	202.5–208.1	KJ01_100	KJ01_112	9.9	−5.23	0.146
	*qGNP3*	3	162.3	158.1–163.3	OUC03_1163	OUC03_1175	7.6	4.73	0.118
	*qGNP5*	5	131.7	129.4–134.1	KJ05_065	KJ05_066	4.5	3.41	0.060
	*qGNP12*	12	93.8	89.7–97.4	KJ12_060	KJ12_061	8.5	−4.99	0.134

CL: culm length; PL: panicle length; PN: panicle number per plant; CD: culm diameter; GNP: grain number per panicle; Chr.: chromosome number; QTL interval: region containing the QTL at 95% probability; LOD: logarithm of the odds score.

## Data Availability

Data are contained within the article and Supplementary Materials.

## Supplementary Materials

The following supporting information can be downloaded at https://www.mdpi.com/article/10.3390/genes14081593/s1: Supplementary Figure S1: Results of QTL analyses of tiller numbers in 2019 and 2022; Supplementary Figure S2: Genotypes of Odae^*5^/Unbong40 BC_4_F_1_ plants at 180 KASP markers; Supplementary Figure S3: Histogram showing distributions of agronomic traits in the F_10_ RIL population derived from a cross between Odae and Unbong40; Supplementary Table S1: Primers used for analysis of the 4 bp TGTG insertion/deletion in the 5’ UTR of *OsTB1*; Supplementary Table S2: Locations of quantitative trait loci (QTL) associated with tiller number identified in the Odae^*5^/Unbong40 BC_4_F_2_:F_3_ population in field trials in 2022; Supplementary Table S3: List of sequence variations between Odae and Unbong40 in the *qTN3* region; Supplementary Table S4: Genotypes of 54 Korean *japonica* varieties at the TGTG insertion/deletion site in the 5’ UTR of *OsTB1*.
