# Supplementary material for: QTL Mapping of Tiller Number in Korean Japonica Rice Varieties
Source: Genes (Basel). 2023 Aug 6;14(8):1593. doi: 10.3390/genes14081593 (PMC10454613; doi:10.3390/genes14081593)
Supplement: Supplementary file 1 [file genes-14-01593-s001.zip › qTN3_supplementary_tables.pdf]

Supplementary Table S1. Primers used for analysis of the 4 bp TGTG insertion/deletion in the 5′ UTR of *OsTB1*.

Gene	MSU ID	RAP ID	Accession No.	Purpose		Primer Sequence (5′→3′)
OsTB1	LOC_Os03g49880	Os03g0706500	AK107083	amplification	Forward	GGAATGGAGGGAGTATGTTTTGTTG
					Reverse	GTGGGTGGTGGTGGTAGTAG
				sequencing	Forward	GAAGAGGATTACAAAGGAGGCA
					Reverse	TGGGGAATCGAAGAAAGGAA
				HRM marker	Forward	CTGCCTCCTAATCCTCCT
					Reverse	ACCATGAGAGAAGAGACCAA

Supplementary Table S2. Locations of quantitative trait loci (QTL) associated with tiller number identified in the Odae*5/Unbong40 BC4F2:F3 population in field trials in 2022.

Measurement Date (DAS)	QTL Name	Chr.	Location (cM)	QTL Interval (cM)	Interval-Flanking Markers		LOD	Additive Effect	R^2
					Left	Right			
2022. 6. 28 (64)	*qTN3*	3	8.91	7.7-10.5	OU3FC_04	OU3FC_07	11.5	1.63	0.268
2022. 7. 5 (71)	*qTN3*	3	8.91	6.1-11.6	OU3FC_01	OU3FC_09	6.0	1.06	0.172

DAS: days after sowing; Chr.: chromosome number; QTL interval: region containing the QTL at 95% probability; LOD: logarithm of the odds score.

Supplementary Table S4. Genotypes of 54 Korean *japonica* varieties at the TGTG insertion/deletion site in the 5′ UTR of *OsTB1*.

Allele Type	Variation in Sequence	Variety
Reference type	CTGTGTGTGTGTTCTACT	Odae, Junam, Hwayoung, Ilpum, Saeilmi, Sodami, Namchan, Samgwang, Nampyeong, Hiami, Dongjin, Hangaru, Heukgwang, Goami, Haedeul, Saenuri, Beakokchal, Asemi, Alchanmi, Yechan, Jopyeong, Younghojinmi, Dodamssal, Sangju, Mangeum, Joun, Geumo, Jinok, Boramchan, Seomyeong, Haedamssal, Seolremi, Daebo, Pyeongwon, Jinbu, Daeribbyeo1ho, Jeokjinju, Cheongpum, Baekjinju1ho, Hopyeong, Aromi (41)
TGTG insertion type	CTGTGTGTGTGTGTGTTCTACT	Unbong40, Dongan, Sindongjin, Ungwang, Heuksujung, Deurechan, Namil, Hyeonpum, Saeodae, Sobi, Huimangchan, Sangnambatbyeo, Boramchal (13)
