# Supplementary material for: QTL Mapping of Tiller Number in Korean Japonica Rice Varieties
Source: Genes (Basel). 2023 Aug 6;14(8):1593. doi: 10.3390/genes14081593 (PMC10454613; doi:10.3390/genes14081593)
Supplement: Supplementary file 1 [file genes-14-01593-s001.zip › qTN3_supplementaty_figures.pptx]

## Slide 1
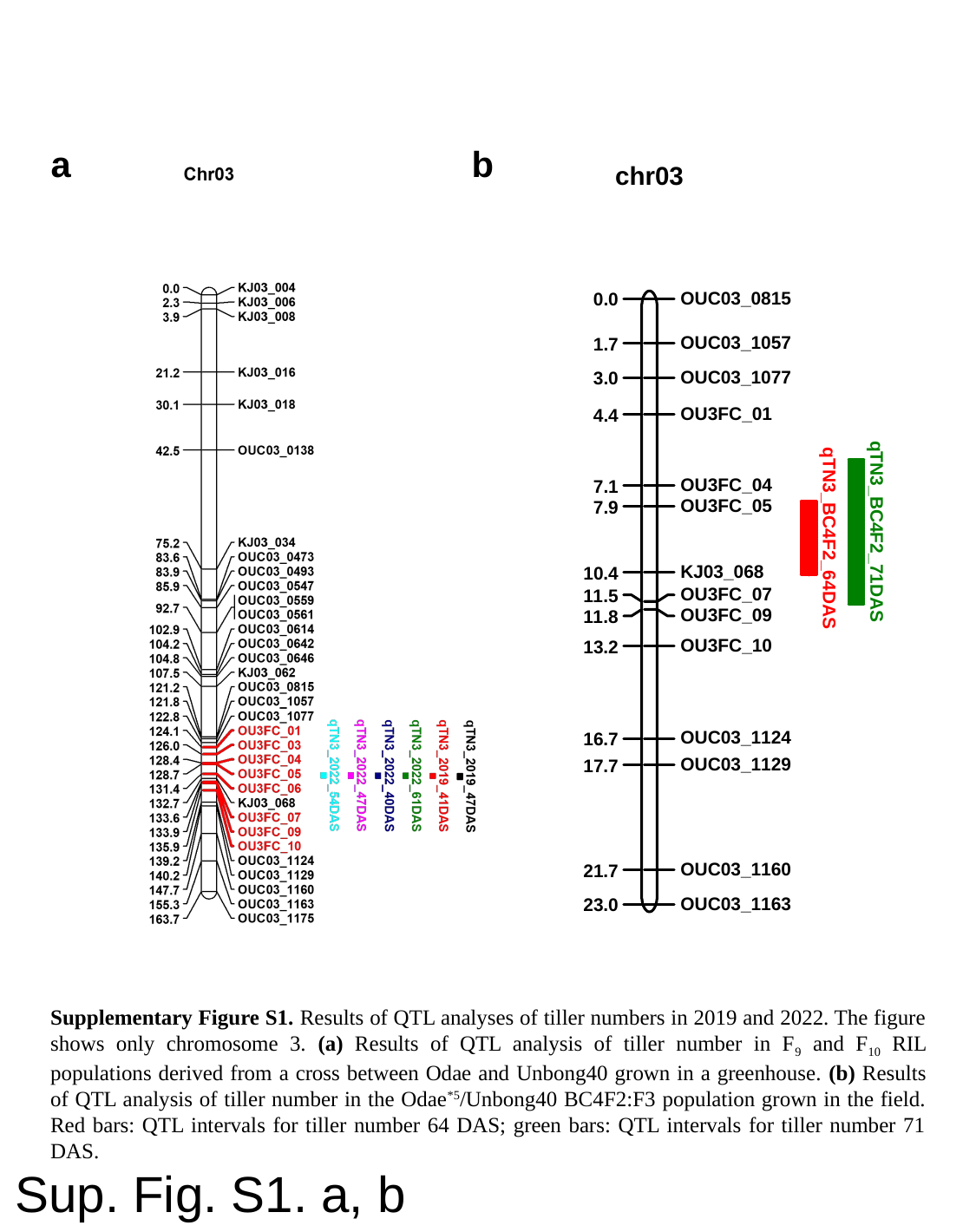

a
b
Supplementary Figure S1. Results of QTL analyses of tiller numbers in 2019 and 2022. The figure shows only chromosome 3. (a) Results of QTL analysis of tiller number in F9 and F10 RIL populations derived from a cross between Odae and Unbong40 grown in a greenhouse. (b) Results of QTL analysis of tiller number in the Odae*5/Unbong40 BC4F2:F3 population grown in the field. Red bars: QTL intervals for tiller number 64 DAS; green bars: QTL intervals for tiller number 71 DAS.
Sup. Fig. S1. a, b

## Slide 2
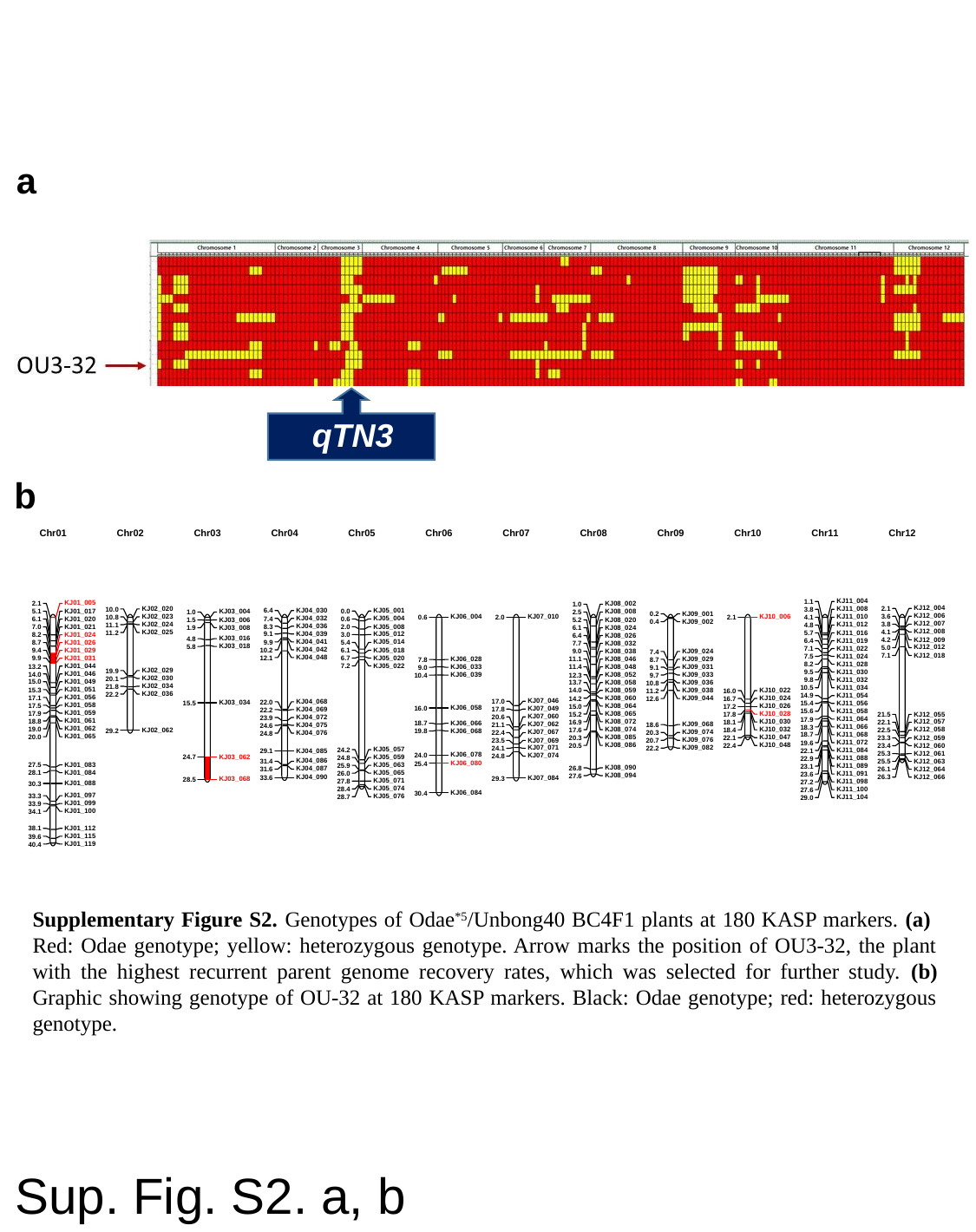

a
qTN3
b
Supplementary Figure S2. Genotypes of Odae*5/Unbong40 BC4F1 plants at 180 KASP markers. (a) Red: Odae genotype; yellow: heterozygous genotype. Arrow marks the position of OU3-32, the plant with the highest recurrent parent genome recovery rates, which was selected for further study. (b) Graphic showing genotype of OU-32 at 180 KASP markers. Black: Odae genotype; red: heterozygous genotype.
Sup. Fig. S2. a, b

## Slide 3
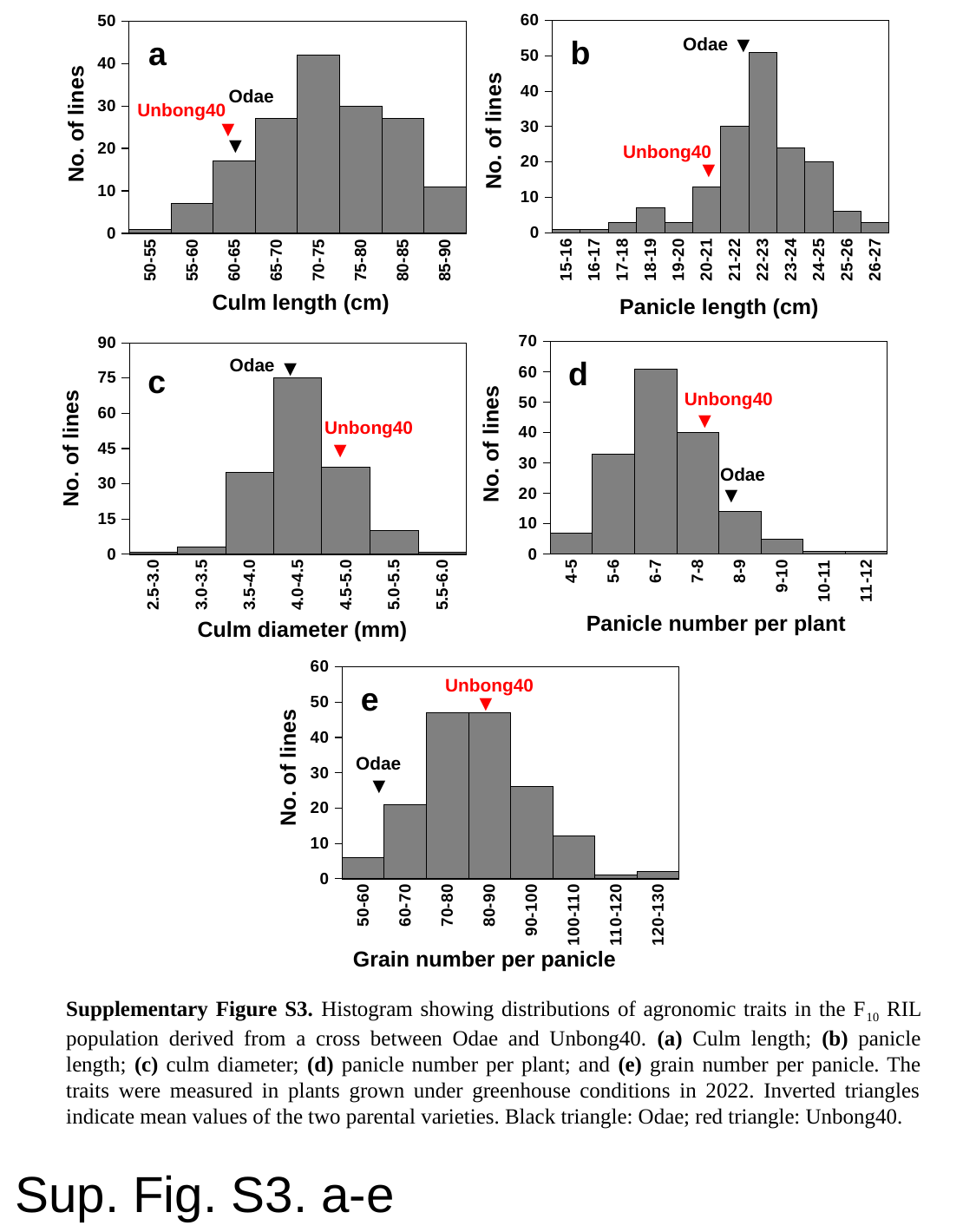

### Chart
| Category | |
|---|---|
| 15-16 | 1.0 |
| 16-17 | 1.0 |
| 17-18 | 3.0 |
| 18-19 | 7.0 |
| 19-20 | 3.0 |
| 20-21 | 13.0 |
| 21-22 | 30.0 |
| 22-23 | 51.0 |
| 23-24 | 24.0 |
| 24-25 | 20.0 |
| 25-26 | 6.0 |
| 26-27 | 3.0 |
### Chart
| Category | |
|---|---|
| 50-55 | 1.0 |
| 55-60 | 7.0 |
| 60-65 | 17.0 |
| 65-70 | 27.0 |
| 70-75 | 42.0 |
| 75-80 | 30.0 |
| 80-85 | 27.0 |
| 85-90 | 11.0 |▼
b
Odae
a
Odae
Unbong40
No. of lines
▼
No. of lines
▼
Unbong40
▼
Culm length (cm)
Panicle length (cm)
### Chart
| Category | |
|---|---|
| 4-5 | 7.0 |
| 5-6 | 33.0 |
| 6-7 | 61.0 |
| 7-8 | 40.0 |
| 8-9 | 14.0 |
| 9-10 | 5.0 |
| 10-11 | 1.0 |
| 11-12 | 1.0 |
### Chart
| Category | |
|---|---|
| 2.5-3.0 | 1.0 |
| 3.0-3.5 | 3.0 |
| 3.5-4.0 | 35.0 |
| 4.0-4.5 | 75.0 |
| 4.5-5.0 | 37.0 |
| 5.0-5.5 | 10.0 |
| 5.5-6.0 | 1.0 |d
Odae
▼
c
Unbong40
▼
Unbong40
No. of lines
No. of lines
▼
Odae
▼
Panicle number per plant
Culm diameter (mm)
### Chart
| Category | |
|---|---|
| 50-60 | 6.0 |
| 60-70 | 21.0 |
| 70-80 | 47.0 |
| 80-90 | 47.0 |
| 90-100 | 26.0 |
| 100-110 | 12.0 |
| 110-120 | 1.0 |
| 120-130 | 2.0 |Unbong40
e
▼
Odae
No. of lines
▼
Grain number per panicle
Supplementary Figure S3. Histogram showing distributions of agronomic traits in the F10 RIL population derived from a cross between Odae and Unbong40. (a) Culm length; (b) panicle length; (c) culm diameter; (d) panicle number per plant; and (e) grain number per panicle. The traits were measured in plants grown under greenhouse conditions in 2022. Inverted triangles indicate mean values of the two parental varieties. Black triangle: Odae; red triangle: Unbong40.
Sup. Fig. S3. a-e
